# A machine learning approach for thermodynamic modeling of the statically measured solubility of nilotinib hydrochloride monohydrate (anti-cancer drug) in supercritical CO_2_

**DOI:** 10.1038/s41598-023-40231-4

**Published:** 2023-08-09

**Authors:** Hassan Nateghi, Gholamhossein Sodeifian, Fariba Razmimanesh, Javad Mohebbi Najm Abad

**Affiliations:** 1 Department of Chemical Engineering, Faculty of Engineering, University of Kashan, Kashan, 87317-53153 Iran; 2 Laboratory of Supercritical Fluids and Nanotechnology, University of Kashan, Kashan, 87317-53153 Iran; 3 Modeling and Simulation Centre, Faculty of Engineering, University of Kashan, Kashan, 87317-53153 Iran; 4 Department of Computer Engineering, Quchan Branch, Islamic Azad University, Quchan, 9479176135 Iran

**Keywords:** Chemical engineering, Chemical engineering

## Abstract

Nilotinib hydrochloride monohydrate (NHM) is an anti-cancer drug whose solubility was statically determined in supercritical carbon dioxide (SC-CO_2_) for the first time at various temperatures (308–338 K) and pressures (120–270 bar). The mole fraction of the drug dissolved in SC-CO_2_ ranged from 0.1 × 10^–5^ to 0.59 × 10^–5^, corresponding to the solubility range of 0.016–0.094 g/L. Four sets of models were employed to evaluate the correlation of experimental data; (1) ten empirical and semi-empirical models with three to six adjustable parameters, such as Chrastil, Bartle, Sparks, Sodeifian, Mendez-Santiago and Teja (MST), Bian, Jouyban, Garlapati-Madras, Gordillo, and Jafari-Nejad; (2) Peng-Robinson equation of state (Van der Waals mixing rule, had an *AARD%* of 10.73); (3) expanded liquid theory (modified Wilson model, on average, the *AARD* of this model was 11.28%); and (4) machine learning (ML) algorithms (random forest, decision trees, multilayer perceptron, and deep neural network with respective R^2^ values of 0.9933, 0.9799, 0.9724 and 0.9701). All the models showed an acceptable agreement with the experimental data, among them, the Bian model exhibited excellent performance with an *AARD%* of 8.11. Finally, the vaporization (73.49 kJ/mol) and solvation (− 21.14 kJ/mol) enthalpies were also calculated for the first time.

## Introduction

Nilotinib hydrochloride monohydrate (NHM) lacks a chiral center, making it incapable of tautomerism. NHM is a chemical compound with the following name: 4-methyl-*N*-[3-(4-methyl-1H-imidazol1-yl)-5-(trifluoromethyl) phenyl]-3-[(4-pyridin-3-ylpyrimidin-2-yl) amino] benzamide hydrochloride monohydrate. It is a white powder with a slight yellowish or greenish-yellowish shade. At 25 °C, the aqueous solubility of NHM markedly decreases with pH. Moreover, it is almost insoluble in buffer solutions with pH values higher than 4.5. NHM shows slight solubility in ethanol and methanol. Further information can be found in Supplementary information.

According to scientific studies, a genetic mutation with unclear reasons in bone marrow hematopoietic (myeloid) cells leads to the formation of a malfunctioning chromosome known as the Philadelphia chromosome^1^. This defective chromosome is present in over 90% of individuals diagnosed with chronic myeloid leukemia (CML). The mentioned genetic abnormality sets in a chain of activities that eventually triggers the growth and reproduction of these cells and their carcinomic progression^2^.

The solubility rate is a critical factor affecting the bioavailability of the active components in orally administered drugs. The poor bioavailability of NHM can be due to its low water solubility, which influences its efficiency in the body. Chemotherapy is a conservative treatment in medical science which involves the use of low doses of drug due to its potential harm to the tumor-adjacent organs. Solubility enhancement avoids the long-term side effects of the drug while considerably reducing the required dosage. Bioavailability increment generally improves the efficacy of the drug in the body^3^. To this end, various techniques such as the utilization of amorphous solid dispersions and RESS can be employed to enhance the bioavailability of the drug. Among these strategies, the reduction of particle size is a prevalent and pragmatic approach.

Particle size reduction is a unique technique in the enhancement of drug solubility. Conventional processes such as sublimation and crystallization have been utilized in the pharmaceutical industry for this purpose. SC-CO_2_ technology is also a promising approach for producing nano-sized and micro-sized particles^4–6^. The use of SC-CO_2_ solvent in industrial plants has been increasing due to its non-toxicity and high effectiveness in extracting compounds. Additionally, it exhibits greater stability in various process and requires lower temperatures compared to alternative solvents. Supercritical fluids (SCFs) are similar to liquids in dissolving power and resemble gases in transfer characteristics (high permeability and low viscosity). The supercritical extraction also enjoys other advantages such as shorter processing time, high selectivity, sensitivity to temperature and pressure variations, and concentration of the solvent to achieve the ideal degrees of freedom for sorting or monitoring the strength of solubility, the sensitivity of the solvent to reach the desired degrees of freedom, better output quality, lower solvent usage, and temperature tolerance for components sensitive to high temperatures^7–9^. SCFs are characterized by their temperature and pressure exceeding the critical temperature (T_c_) and critical pressure (P_c_), respectively, enabling them to exhibit the properties of both a liquid and a gas. In the proximity of critical temperatures, SCFs demonstrate substantial compressibility, which facilitates moderate variations in pressure density and mass transport features that significantly influence their solvent capacity. Furthermore, the environmental concerns of toxic solvents can be resolved by the use of CO_2_ gas as a solvent in supercritical procedures due to its neutrality. As an example, organic solvents, particularly chlorinated solvents, are highly hazardous to the environment. Chlorinated solvents and several other industrial solvents, such as chlorofluorocarbons, have been shown to be harmful for the ozone layer. The substitution of chlorofluorocarbon with CO_2_ is an instance of resolving the issue of toxic and polluting solvents through the use of CO_2_ solvent. Other advantages of SC-CO_2_ process include its cost-effectiveness and availability at high purity and low critical pressure and temperature (P_C_ = 73.8 bar and T_C_ = 304.18 K), chemical neutrality desirable for use in food and pharmaceutical industries, suitability in critical conditions, and non-toxicity and non-flammability^10^. The researchers select the temperature and pressure based on previous studies and the constraints of the laboratory equipment^8,11,12^.

The solubility data presented in this study are valuable for evaluating the applicability of SCFs in a specific industrial process. Additionally, these data can aid in identifying the optimal conditions, in terms of temperature, pressure, and experimental measurement technique, for effective use of these fluids. Knowledge regarding the solubility of medicinal substances and their correlation with thermodynamic models in SC-CO_2_ can help in the development of pharmaceutical processes. This is due to the fact that the determination of solubility under various temperature conditions and high pressures can be monetarily costly and time-intensive.

As mentioned before, the experimental measurement of solubility in SC-CO_2_ is a valuable but time-consuming, complex, and costly task. Therefore, there is a need for alternative approaches, such as computational techniques, to replace experimental assessments. To this end, several theoretical methods have been proposed and developed for predicting the solubility of drugs in SC-CO_2_. Several situations influence molecular characteristics in liquid systems, which are connected to all nearby molecules in motion. Describing these specifications needs an efficient experiment and thermodynamic modeling of all potential solvent and soluble molecule configurations^7,13^. Empirical and semi-empirical, equations of state (EoSs), solid–liquid equilibrium models, and machine learning algorithms are the four most popular forms of thermodynamic models, each with a unique variety of ranges and notable scopes. Modeling with experimental and semi-empirical approaches requires no definition of the critical characteristics. These models rely on the SCF temperature, pressure, and volumetric mass. Models with three to six adjustable parameters, such as Bian^14^, Chrastil^15^, Jafari-Nejad^16^, Jouyban^17^, Sparks^18^, Sodeifian^12^, Mendez-Santiago and Teja^19^, Garlapati-Madras^20^, Bartle^21^, and Gordillo^22^ offer proper analysis for evaluating the reliability of experimental data. Different mixing rules are also employed in EoS models which enjoy applicability across a wide range of temperatures and pressures for fluids with various densities, from low-density gases to dense liquids. These equations are also applicable to gases, liquids, and SCFs. In addition, activity coefficient models, such as ELT, are commonly used to model solubility in SCFs. This approach assumes that the fugacity of the solid phase is equivalent to the SCF^23^.

Machine learning refers to a constantly developing group of computing instructions to simulate intelligence by learning from the surroundings. This strategy is now regarded as a means of communication with big data in the modern era. Machine learning-based algorithms have been successful in diverse fields, including pattern recognition, biomedical and medical applications, spacecraft engineering, computational biology, financial sectors, and entertainment^24^. Ionizing radiation (radiotherapy) is used to treat more than half of cancer cases; it is also the primary method in the treatment of advanced stages of localized diseases. Radiation therapy involves a variety of steps that extend not just from consultation through treatment but even beyond. Machine learning should be used to guarantee that patients receive the appropriate quantity of radiation and respond properly to the therapy. These instructions are easily programmable as they naturally modify their structure via repetitions (i.e., experience) for better production of the desired output. An effective approach presents two primary benefits: the ability to supplant laborious and repetitive human duties, and more importantly, capability of detecting complex patterns of incoming data which exceeds the ability of an average human observer. The significance of these benefits is particularly in radiation therapy. However, given the limitations of this approach, the final results are prone to uncertainty and observer variability. An imaging guide can identify microscopic features of an organ, immediately synthesizing information from several sources, or combining the knowledge of multiple observers to achieve low imaging error.

This research presents the first reports on the solubility of NHM in SC-CO_2_ within the temperature and pressure ranges of 308–338 K and 120–270 bar, respectively. Besides, four types of models were used to evaluate the correlation of the empirical findings of NHM: (1) ten empirical and semi-empirical density-based models (Chrastil, Bartle, Sparks, Sodeifian, MST, Bian, Jouyban, Garlapati-Madras, Gordillo, and Jafari-Nejad), (2) PR EoS model with vdW mixing rule, (3) ELT model (modified-Wilson model) for correlating fugacity, (4) ML algorithms (RF, DTs, MLP, and DNN) with 17 solubility datasets available in the previously published papers. The validity assessment of multiple models involved the evaluation of deviations of computed outcomes from empirical solubility data, utilizing three actual measures: average absolute relative deviation (*AARD%*), adjusted correlation coefficient (R_adj_), and F-value.

## Experimental

### Materials

NHM was supplied from Parsian Pharmaceutical Company with a guaranteed purity of 99% (Tehran-Iran) while CO_2_ (purity > 99.99%) was provided by Fadak Company (Kashan, Iran). Analytical grade dimethyl sulfoxide (DMSO) was also purchased from Merck (Darmstadt, Germany). Table 1 lists the physical and chemical characteristics of NHM.

**Table 1:** Molecular structure and physicochemical specifications of NHM.

Compound	Formula	Structure	M_w_ (g/mol)	T_m_ (K)	λ_max_ (nm)	CAS number	Minimum purity	Producer
Nilotinib hydrochloride monohydrate	C_28_H_25_ClF_3_N_7_O_2_		584	480.15 ± 7	270	923288-90-8	99%(HPLC)	Parsian Pharmapeutical Co (Tehran, Iran)
Carbon Dioxide	CO_2_		44.01	–	–	124-38-9	99.99%(GC)	Fadak Co (Kashan, Iran)
Dimethyl sulfoxide	C_2_H_6_OS		78.13	–	–	67-68-5	99%(GC)	Merck Group (Darmstadt, Germany)

### Experimental apparatus

**Figure 1:**
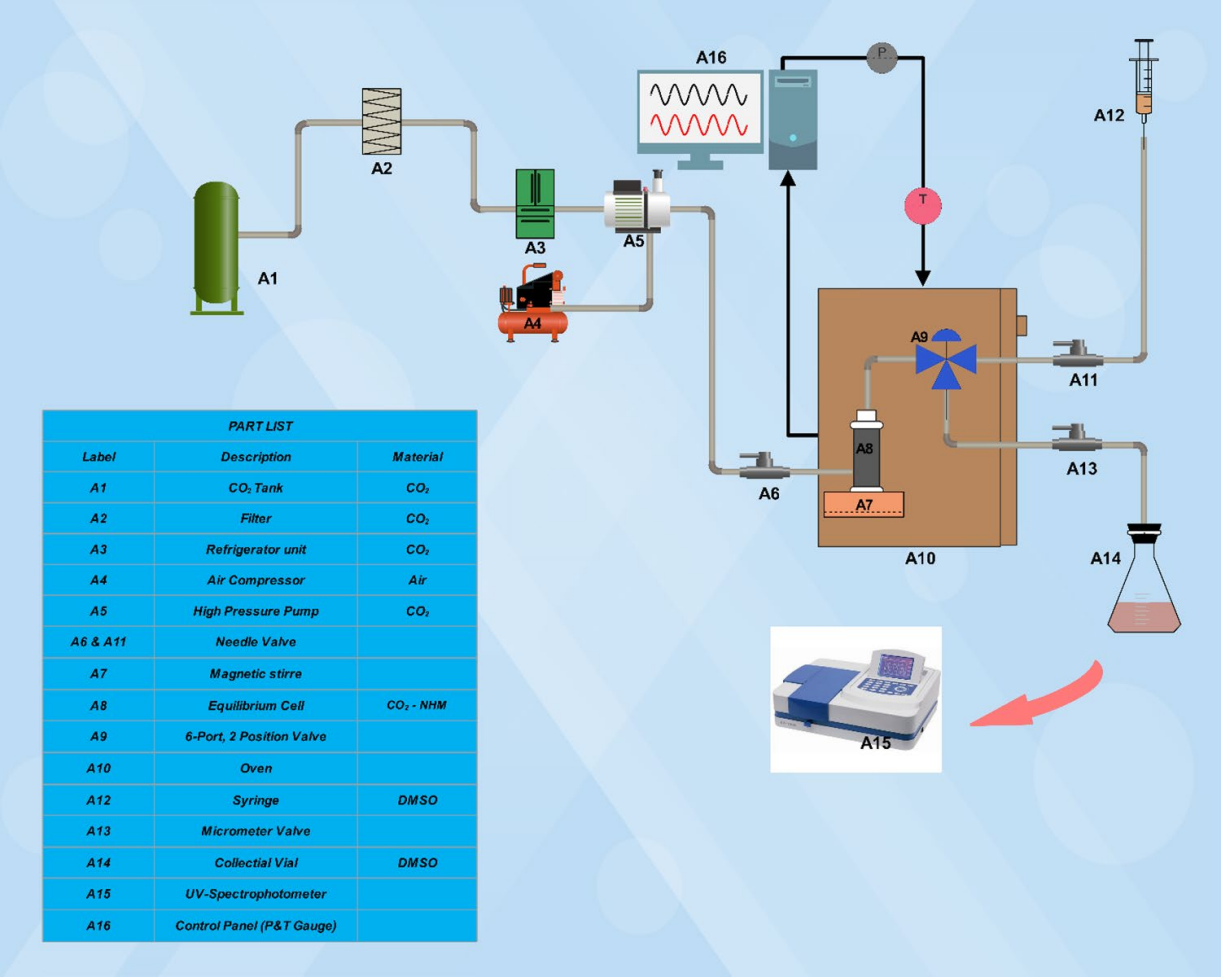
Schematic of the used laboratory equipment for measuring solubility.

This work aimed to determine the equilibrium solubility of NHM using a static approach. To this end, a UV–Vis spectrophotometer was utilized along with the equipment described in our previous research^13,25–27^. The experimental setup comprised various components, including gas cylinders, filters, sampler, refrigeration, heating elements, flow meter, 6-way valve, and a micrometer valve. All valves, connections, and piping were 1/8″ in size. The process is fully described in the previous work of the authors; however, a quick review is also provided here. Figure 1 depicts the experimental setup.

The operation began with liquefying CO_2_ (the required temperature for CO_2_ liquefication ranges between − 15 to – 20 °C), which is an important section in CO_2_ preparation for pumping. A pressure gauge was employed to monitor pressure with an accuracy of ± 1 bar (EN 837-1, WIKA, Germany), while an oven was used to maintain the temperature of CO_2_ around the desired temperature within a tolerance of ± 0.1 K. Then, NHM (1 g) was placed inside a 70-mL equilibrium tube with 2 mm glass beads and sintering filters (1 µm, keeps undissolved drugs in the container) on both surfaces. The glass beads were used to homogeneously mix the drug and reduce channeling to enhance the interaction areas between the sample and SCF, following this, the container was exposed to SC-CO_2_. Based on previous publications, the present study achieved the equilibrium state within 60 min of equilibration^11,13,25,28^. Afterward, 600 µL of SC-CO_2_ (at saturation level) was introduced to the injection cycle through a 6-port, 2-position valve. Upon releasing the injection valve, the aforementioned substance moved toward the gathering vial, which had been already filled with a specific quantity of DMSO. Subsequently, the gathered DMSO (1 mL) in the vial was employed to clean the cycle. The total volume of the solution was 5 mL. The solubility of NHM in SC-CO_2_ was determined through maximum absorption observations, using a double-beam UV–Vis spectrophotometer (Unico, SQ-4802, USA) and 1-cm route silica cells. The solid pharmaceutical was dissolved in DMSO to achieve a final concentration of 100 μg L^−1^. Various dilutions were obtained by utilizing the standard solution within acceptable limits. The condensation of the drug in the collecting vial was determined using a standard curve with a regression ratio of 0.99. The UV spectral data were collected at 270 nm on a UV–Vis spectrophotometer to investigate NHM^29^. The equilibrium values of mole fraction (y_2_) and solubility (S (g/L)) in SC-CO_2_ were computed for various pressures and temperatures, using the following equations:

1 $${y}_{2}=\frac{{n}_{solute}}{{n}_{solute}+{n}_{C{O}_{2}}},$$

where:

2 $${n}_{solute}=\frac{{C}_{s}(\frac{g}{L}){V}_{s}(L)}{{M}_{s}(\frac{g}{mol})},$$

3 $${n}_{C{O}_{2}}=\frac{{V}_{l}\left(L\right)\rho \left(\frac{g}{mol}\right)}{{M}_{C{O}_{2}}\left(\frac{g}{mol}\right)}.$$

In which, ${n}_{solute}$ and ${n}_{C{O}_{2}}$ show the moles of solute (NHM) and CO_2_ in the measurement cycle, respectively. ${C}_{s}$ is the solute content (g/L) in the gathering vial according to the standard curve; while ${V}_{s}(L)$ = 5 × 10^–3^ and ${V}_{l}\left(L\right)$ = 600 × 10^–6^ denote the volume of the gathering vial and measuring cycle, respectively. ${M}_{s}$ and ${M}_{C{O}_{2}}$ also represent the molecular weights of the solute and CO_2_. Equation (4) was used to estimate the equilibrium solubility of the solute, S (g/L), in SC-CO_2_^30^.

4 $$S\left(\frac{g}{L}\right)=\frac{{C}_{s}(\frac{ g}{L}){V}_{s}(L)}{{V}_{l}(L)}.$$

## Theoretical background

In this work, the solubility of NHM was correlated with four types of models: (1) Empirical and semi-empirical models such as Chrastil, Bian, Sodeifian, etc. with 3–6 adjustable parameters; (2) EoS based model like Peng-Robinson with vdW mixing rule; (3) ELT (modified Wilson model) and (4) ML algorithms such as random forests, decision trees, MLP, and DNN. Details of the mentioned models can be found in the continue.

### EoS-based model

The solubility of a solid solute in a SC-CO_2_ can be expressed as follows:

5 $${y}_{2}=\frac{{P}_{2}^{s}{\phi }_{2}^{s}}{P{\phi }_{2}^{f}}\mathrm{exp}\left[\frac{\left(P-{P}_{2}^{s}\right){\upsilon }_{2}}{RT}\right],$$

where *P* shows the pressure at the system temperature, ${P}_{2}^{s}$ denotes the pressure required for the sublimation of the pure solute. The molar volume of the solute is also represented by ${\upsilon }_{2}$, while the universal gas constant is denoted by R. Furthermore, the pure solute fugacity coefficient, denoted by ${\phi }_{2}^{s}$, is assumed to be 1.

In the present research, PR-EoS^13,25,28,31^ can be described as follows^32,33^:

6 $$P=\frac{RT}{\upsilon -b}-\frac{a\left(T\right)}{\upsilon \left(\upsilon +b\right)+b\left(\upsilon -b\right)}.$$

vdW2 is represented as:

7 $$a={y}_{1}^{2}{a}_{11}+{y}_{2}^{2}{a}_{22}+2{y}_{1}{y}_{2}{a}_{12},$$

8 $$b={y}_{1}^{2}{b}_{11}+{y}_{2}^{2}{b}_{22}+2{y}_{1}{y}_{2}{b}_{12}.$$

In addition, Table 1S (supplementary information) presents a summary of the EoS-based model.

9 $${a}_{12}=\left(1-{k}_{12}\right)\sqrt{{a}_{11}{a}_{22}},$$

10 $${b}_{12}=\left(1-{l}_{12}\right)\frac{\left({b}_{11}+{b}_{22}\right)}{2}.$$

${k}_{12}$ and ${l}_{12}$ are interaction parameters whose values were determined by an objective function (OF) aimed at minimizing the resulting output. Thus, ${k}_{12}$ and ${l}_{12}$ can be obtained through the minimization of the mentioned objective function.

11 $$OF=\sum_{i=1}^{N}\frac{\left|{y}_{2,i}^{exp}-{y}_{2,i}^{calc}\right|}{{y}_{2,i}^{exp}}.$$

### ELT model

The ELT takes SCFs as expanded liquids since their density is closer to that of a liquid rather than a gas. Therefore, this theory establishes thermodynamic phase equilibrium between solute and SCF as the solid–liquid equilibrium relevance and contractual activity coefficients. Accurate estimation of solid solubility in the supercritical (SC) phase relies on proper knowledge on these activity coefficients, which can be determined by understanding the fugacity of the composites. When the equilibrium and the fugacity are equivalent in two steps, the coefficients can be obtained by^34^:

12 $${f}_{2}^{s}={f}_{2}^{L}.$$

Here, the Modified Wilson model was employed to determine the activity coefficient of the solid solute.

Furthermore, the dimensionless energies of interaction can be determined by:

13 $$\lambda^{\prime}_{12} = \frac{{\lambda_{12} }}{{RT_{c} }},$$

14 $$\lambda^{\prime}_{21} = \frac{{\lambda_{21} }}{{RT_{c} }}.$$

An empirical expression was introduced to modify the Wilson model, considering the impact of high pressures. The modified model facilitates the prediction by establishing a linear correlation between the molar volume and density reduction.

15 $${\upsilon }_{2}=\alpha {\rho }_{r}+\beta .$$

The regressed parameters of the model are given by α, β, $\lambda^{\prime}_{12} ,$ and $\lambda^{\prime}_{21}$. Detailed information is available in supplementary information. Genetic algorithm (GA), nonlinear regression^35^ and simulated annealing (SA) algorithms were utilized to obtain optimum double interaction and regressed parameters of the ELT.

### Semi-empirical models

The solubility data of NHM in SC-CO_2_ were compared with ten semi-empirical models. Researchers such as Garlapati-Madras, Chrastil, Bartle, MST, Sparks, Jouyban, Bian, and Sodeifian worked in this field and presented different models, as listed in Table 2.

**Table 2:** Summary of empirical models applied in present work.

Model	Formula	References
Chrastil	$c={\rho }^{{a}_{0}}\mathrm{exp}(\frac{{a}_{1}}{T}+{a}_{2})$	^15^
Bian	${y}_{2}= {\rho }^{({a}_{0}+{a}_{1}\rho )}\mathrm{exp}(\frac{{a}_{2}}{T}+\frac{{a}_{3}\rho }{T}+{a}_{4})$	^14^
Gordillo	$\mathrm{ln}y={a}_{0}+{a}_{1}P+{a}_{2}{P}^{2}+{a}_{3}PT+{a}_{4}T+{a}_{5}{T}^{2}$	^22^
Jafari-nejad	$\mathrm{ln}y={a}_{0}+{a}_{1}{P}^{2}+{a}_{2}{T}^{2}+{a}_{3}\mathrm{ln\rho }$	^16^
Garlapati-Madras	$ln{y}_{2}={a}_{0}+\left({a}_{1}+{a}_{2}\rho \right)ln\rho +\frac{{a}_{3}}{T}+{a}_{4}\mathrm{ln}(\rho T)$	^20^
MST	$Tln\left({y}_{2}P\right)={a}_{0}+{a}_{1}\rho +{a}_{2}T$	^19^
Jouyban	$ln{y}_{2}={a}_{0}+{a}_{1}\rho +{a}_{2}{P}^{2}+{a}_{3}PT+\frac{{a}_{4}T}{P}+{a}_{5}\frac{ln\rho }{T}$	^17^
Sparks	${c}_{2}^{*}={\rho }_{r,1}^{{a}_{0}+{a}_{1}{\rho }_{r,1}}\mathrm{exp}({a}_{2}+\frac{{a}_{3}}{T})$	^18^
Bartle	$\mathrm{ln}\left(\frac{{y}_{2}P}{{P}_{ref}}\right)={a}_{0}+\frac{{a}_{1}}{T}+{a}_{2}(\rho -{\rho }_{ref})$	^21^
Sodeifian	$ln{y}_{2}={a}_{0}+{a}_{1}\frac{{P}^{2}}{T}+{a}_{2}\mathrm{ln}\left(\rho T\right)+{a}_{3}\left(\rho ln\rho \right)+{a}_{4}P lnT+{a}_{5}\frac{ln\rho }{T})$	^12^

The discussion is focused on evaluating several models based on their *AARD%*, R_adj_, and F-value to identify the models with acceptable accuracy. The least squares method (LSM) was utilized for calculating curve-fitting variables. *AARD%* was utilized as a criterion to ensure comparable analyses, since the number of curve-fitting parameters is closely related to correlation precision. The value of *AARD%* was determined by the following equation, where Z represents the number of curve-fitting variables for the given model^36,37^:

16 $$AARD\%=\frac{100}{{N}_{i}-Z}\sum_{i=1}^{{N}_{i}}\frac{\left|{y}_{2}^{calc}-{y}_{2}^{exp}\right|}{{y}_{2}^{exp}}.$$

The models were further evaluated by a criterion known as R_adj_ with the following definition:

17 $${R}_{adj}=\sqrt{\left|{R}^{2}-\left(Q\left(1-{R}^{2}\right)/\left(N-Q-1\right)\right)\right|}.$$

In the above equation, "*N*" denotes the number of sample points within each set. "*Q*" refers to the number of self-determining changeable elements in each equation and "*R*^2^" represents the correlation analysis^36,38^. F-value is another criterion in the assessment of the capacity of the models to match solubility data, which can be described as follows^39^.

18 $$F{\text{-}}value=\frac{\frac{{SS}_{R}}{Q}}{\frac{{SS}_{E}}{\left(N-Q-1\right)}}=\frac{{MS}_{R}}{{MS}_{E}}.$$

As seen, SS_T_ indicates the total of square summation, SS_R_ represents the sum of squares of the regression, while MS_R_ pertains to the average square of regression. Furthermore, MS_E_ concerns with the average square of residuals. The F-value operates similar to the distribution and is characterized by *Q* and *N-Q-1* grades of independence.

### ML algorithms

Four ML algorithms were employed in this work to examine and evaluate the solubility of NHM. To this end, 432 data samples were used including data of 17 other drugs (published in the literature). These 17 drugs are described in Table 3.

**Table 3:** Solubility data sets used in this work.

Drug name	Structure	Data points	Temperature range (K)	Pressure range (bar)	References
Amlodipine besylate	C_26_H_31_ClN_2_O_8_S	24	308–338	120–270	^36^
Azathioprine	C_9_H_7_N_7_O_2_S	24	308–338	120–270	^31^
Clemastine fumarate	C_25_H_30_ClNO_5_	24	308–338	120–270	^10^
Dasatinib monohydrate	C_22_H_28_ClN_7_O_3_S	24	308–338	120–270	^25^
Empagliflozin	C_23_H_27_ClO_7_	24	308–338	120–270	^39^
Imatinib mesylate	C_30_H_35_N_7_O_4_S	24	308–338	120–270	^12^
Losartan potassium	C_22_H_22_ClKN_6_O	24	308–338	120–270	^13^
Metochloropramide HCl	C_14_H_23_Cl_2_N_3_O_2_	24	308–338	120–270	^28^
Pantoprazole sodium sesquihydrate	C_32_H_34_F_4_N_6_Na_2_O_11_S_2_	24	308–338	120–270	^40^
Pholcodine	C_23_H_30_N_2_O_4_	24	308–338	120–270	^7^
Prazosin HCl	C_19_H_22_ClN_5_O_4_	24	308–338	120–270	^8^
Quetiapine hemifumarate	C_21_H_25_N_3_O_2_S	24	308–338	120–270	^9^
Sorafenib tosylate	C_28_H_24_ClF_3_N_4_O_6_S	24	308–338	120–270	^41^
Sulfabenzamide	C_13_H_12_N_2_O_3_S	24	308–338	120–270	^42^
Sunitinib malate	C_26_H_33_FN_4_O_7_	24	308–338	120–270	^43^
Triflunomide	C_12_H_9_F_3_N_2_O_2_	24	308–338	120–270	^44^
Palbociclib	C_24_H_29_N_7_O_2_	24	308–338	120–270	^45^

Seventy-five percent of the samples were used for training (324), while 25% of them (108) were used for testing. Six basic parameters, including operating temperature and pressure, CO_2_ density, fusion enthalpy, fusion temperature, and sublimation pressure, were taken as effective factors of solubility. Drug solubility is influenced by various factors, among which, temperature, pressure, and CO_2_ density have been identified as key factors in numerous experimental and semi-experimental relationships. Solubility decreases by enhancing molecular weight due to the higher fusion enthalpy and temperature. Additionally, an increase in sublimation pressure results in greater solubility. This data set was evaluated by four algorithms: random forest, decision trees, multilayer perceptron, and deep neural networks. A summary of the applied algorithms can be found below:

#### Decision trees (DTs)

Data mining refers to a vast field of research dealing with pattern identification and categorization of massive and unclear data, in various formats such as text, audio, and video. Sometimes the presented data are insufficient, noisy, or destroyed. One strategy for dealing with this sort of data is classification. Decision trees are employed in data discovery and machine learning to provide an approximate answer. The DT algorithm is a highly effective and powerful tool for data mining capable of handling diverse input data, including nominal, numerical, and alphabetical which is one of the strengths of this algorithm.

This method is capable of processing incomplete data which encompass errors through various platforms and available data packages. DTs extract data from a huge deal of accessible information using decision rules. A DT merely categorizes data to be readily saved and classed again^46,47^.

#### Random forest (RF)

The RF algorithm is a sort of ensemble learning that can be utilized for both assortment and regression test. RF was first developed by Breiman to combine his bagged sampling methodology with the random selection of features^48^ originally described by Ho^49^, Amit and Garden^50^. This approach results in a set of decision trees with controlled variance. Bagging is used to randomly select training data with replacement to construct each tree. Studies have shown that approximately 64% of all occurrences will be represented within this selection. The residual samples (near 36%) are considered out-of-bag samples. In the RF model, each tree operates as a classification algorithm and specifies the class tag of an untagged sample using majority verdict^51^. Each classifier generates a model of its vote for the class tag it expects, and the tag with the maximum votes will be chosen as the category of the sample. Further information is available in supplementary section.

#### Multilayer perceptron (MLP)

The MLP can be classified as a feedforward and fully connected artificial neural network (ANN). It commonly refers to any feedforward ANN and sometimes a network consisting of multiple perceptron layers which may raise the confusion. In cases with only one hidden layer, the multilayer perceptron is often referred to as a "vanilla" neural network. The MLP includes at least three node substrates: entry, undercover, and output substrates. Apart from the input nodes, each node represents a neuron with a nonlinear activation duty. A supervised learning approach labeled as backpropagation is utilized to train the MLP. The MLP differs from the linear perceptron in its use of multiple layers and nonlinear activation, enabling the identification of data which cannot be separated linearly^52^.

If all neurons in a MLP utilize a linear activation function to connect the weighted inputs to the output of every neuron, any number of layers can be reduced to a two-layer input–output model using linear algebra. However, some neurons in MLPs utilize a nonlinear activation function that imitates the modulation of action possibilities or firing in actual neurons^53^.

#### Deep neural network (DNN)

The deep neural network (DNN) is a neural network with a significant number of layers, known as "deep" layers. Utilizing advanced algorithms and structures, the DNN model can be considered a variant of the multilayer perceptron neural network (MLP). Comprising many layers of nodes, DNN is arranged using algorithms to extract deputations from datasets with no need for manual design of feature extractors. As its name implies, deep learning has a larger or deeper number of processing layers compared to the shallow learning with less units. The transition from surface to deep learning enables the planning of more complicated and nonlinear functions, which could not be efficiently mapped using external architectures. Various designs have addressed the difficulties in multiple fields or applied cases^54,55^.

## Results and discussion

### Experimental data

**Figure 2:**
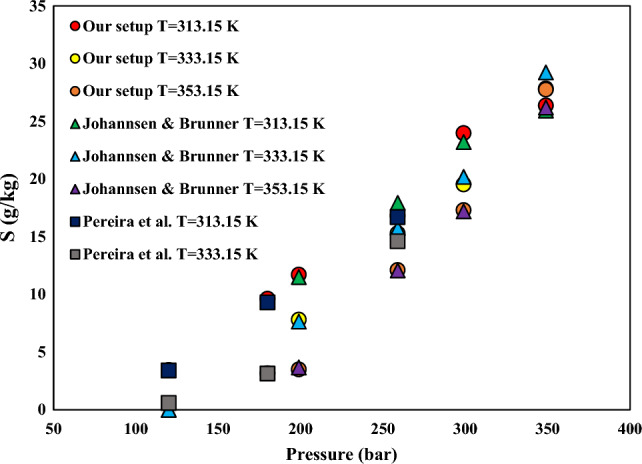
Solubility S (g/kg) of α-tocopherol in CO_2_: Our setup, Pereira et al.^62^ and Johannsen and Brunner^61^ all are shown by symbols.

The reliability and validity of the solubility system and experimental outcomes were assessed in a recent article which evaluated the solubility of Riluzole in SC-CO_2_^56–58^. Examination of the methods and equipment utilized in the experiments requires evaluation of the solubility of a "prototype solute"^59^. Our prior research involved measuring the solubility of naphthalene and α-tocopherol, which was then compared to previously reported data to confirm the dependability of both the apparatus and experimental findings^31,57,60^. Furthermore, the apparatus underwent a secondary validation process utilizing α-tocopherol in a CO_2_ environment prior to drug solubility measurements whose validation outcomes are illustrated in Fig. 2. The current investigation exhibits a satisfactory consistency with other references^61,62^. In this work, the equilibrium solubility S (g/L) and related mole fraction values of NHM were calculated at several pressures and temperatures (120–270 bar and 308–338 K, respectively) as summarized in Table 4.

**Table 4:** Solubility of NHM in SC-CO_2_ at different temperatures and pressures. The experimental standard deviation was calculated by ${u}_{combined}/{y}=\sqrt{\sum_{i=1}^{N}{\left({P}_{i}u\left({x}_{i}\right)/{x}_{i}\right)}^{2}}$.Expanded uncertainty (U) and the relative combined standard uncertainty (u_combined_/y) are determined, respectively, as follows: (U) = k*u_*combined*_(k = 2) and $u_{combined}/y=\sqrt{\sum_{i=1}^{N}{\left({P}_{i}u\left({x}_{i}\right)/{x}_{i}\right)}^{2}}$. In this work, *u*(*x*_*i*_) was considered as standard uncertainties of temperature, pressure, mole fraction, volumes and absorption. P_i_, sensitivity coefficients, are equivalent to the partial derivatives of y equation (Eq. 1) with respect to the ***x***_***i***_.

Temperature (K)^a^	Pressure (bar)^a^	Density (kg/m^3^)^b^	y_2_ × 10^5^ (mole fraction)	S (g/L)	Standard deviation of the mean, SD (ȳ) × 10^5^	Expanded uncertainty of mole fraction (10^5^ U)
308	120	769	0.104	0.016	0.002	0.006
308	150	817	0.125	0.02	0.001	0.006
308	180	849	0.151	0.025	0.002	0.007
308	210	875	0.195	0.034	0.001	0.009
308	240	896	0.219	0.039	0.003	0.011
308	270	914	0.27	0.049	0.002	0.012
318	120	661	0.071	0.009	0.002	0.005
318	150	744	0.141	0.021	0.002	0.007
318	180	791	0.199	0.031	0.002	0.009
318	210	824	0.228	0.037	0.003	0.011
318	240	851	0.269	0.046	0.003	0.013
318	270	872	0.313	0.054	0.004	0.016
328	120	509	0.051	0.005	0.002	0.004
328	150	656	0.191	0.025	0.001	0.008
328	180	725	0.239	0.034	0.002	0.011
328	210	769	0.313	0.048	0.003	0.015
328	240	802	0.433	0.069	0.004	0.02
328	270	829	0.514	0.085	0.004	0.024
338	120	388	0.032	0.002	0.001	0.002
338	150	557	0.252	0.028	0.001	0.011
338	180	652	0.355	0.046	0.003	0.017
338	210	710	0.424	0.06	0.004	0.021
338	240	751	0.563	0.084	0.004	0.026
338	270	783	0.599	0.094	0.006	0.029

^a^Standard uncertainty u are u(T) =  ± 0.1 K; u(p) =  ± 1 bar. The value of the coverage factor k = 2 was selected according to the level of confidence of almost 95 percent for computing the expanded uncertainty.

^b^CO_2_ density, is taken from NIST chemistry web-book (http://webbook.nist.gov/chemistry/).

**Figure 3:**
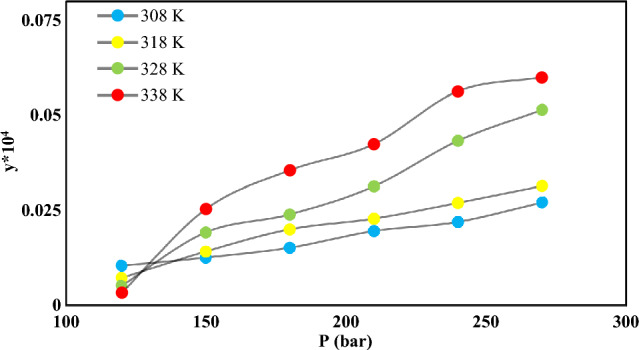
NHM solubility in SC-CO_2_ vs. pressure.

Noteworthy, the data points were measured three times to ensure the reliability and maintain the relative standard uncertainties below 5%. Further information on the mole fractions uncertainties can be found in Table 5. The Span-Wagner equation (a CO_2_-specific EoS) was also employed to define the density of SC-CO_2_^63^. The mole fraction (y) and solubility (S (g/L)) values of NHM ranged from 0.1 × 10^–5^ to 0.59 × 10^–5^ and 0.016–0.094, respectively. The highest and lowest values for the solubility of the solid medicine were detected at 338 and 338 K and pressures of 270 and 120 bar, respectively. The fundamental mechanism of drug solubility entails the disruption of intermolecular or inters ionic bonds among solute molecules. This provides enough room for solvent molecules to penetrate the solute molecules and facilitates particle wetting, which enables the required solvent–solute interactions for dissolution. As illustrated by the isotherms in Fig. 3, the solubility of NHM increased by enhancing the pressure at fixed temperatures. At a specific temperature, an increase in pressure leads to greater gas dissolution in a solvent, whereas a decrease in pressure reduces the gas solubility in a liquid. For example, when producing carbonated drinks, additional pressure is applied to the solute to enhance the solubility of CO_2_ in the liquid. The pressure enhancement compresses gas molecules within the solute, which creates more space for extra gas molecules, increasing the solubility of CO_2_ in the liquid. Therefore, the solubility of gases in liquids increases with pressure increment. From another point of view, when a gas molecule in the vapor phase makes contact with a liquid surface, it can either be repelled back into the gas or dissolved into the liquid to become a solute particle. Upon reaching the liquid surface, dissolved molecules will gain enough kinetic energy to escape into the gas phase. Therefore, there will be a constant exchange of particles across the gas–liquid boundary. Equilibrium is achieved at equal entry and exit rates of the gas phase, resulting in constant concentrations in each phase. Solubility is a measure of the concentration of dissolved gas particles in the liquid and is dependent on gas pressure. An increase in pressure leads to an increment in the collision frequency, increasing the solubility. Conversely, a decrease in pressure decrements the solubility. Such an increase in solubility can be assigned to the enhanced density and improved solvating power of SC-CO_2_ at higher pressures. The temperature has a dual impact on the solubility in SC-CO_2_ depending on the variations of vapor pressure of the solute and solvent density. The effect of temperature on solubility is contingent upon the characteristics of both the solute and solvent, including their interactions; while the behavior of solid and gaseous solutes differs. Solid solutes show solubility increment with raising the temperature, whereas gas solutes tend to become less soluble. This effect can be attributed to the heightened kinetic energy that is accompanied by an increase in temperature. Specifically, gas molecules possess greater kinetic energy at higher temperatures, which promotes the dissociation of intermolecular bonds between the gas solute and solvent. An increment in the solution temperature can augment the vapor pressure of the solute, further enhancing the solvation ability of SCF^60,64,65^.

**Table 5:** *AARD%* and correlation parameters of empirical and semi-empirical models for NHM solubility in SC-CO_2_.

Model	*a*_0_	*a*_1_	*a*_2_	*a*_3_	*a*_4_	*a*_5_	*AARD%*	R_adj_	F-value
Chrastil	5.2519	− 6296.2307	− 19.0748	–	–	–	12.29	0.9688	118.37
Bian	4.3527	− 0.0077	− 21,319.037	19.0798	18.1565		8.11	0.9763	235.17
Gordillo	− 28.9404	− 0.7702	− 0.0084	0.0038	0.1243	− 0.0003	20.92	0.9321	51.79
Jafarinejad	− 20.936	0.000005	0.000084	3.9564	–	–	11.6	0.9581	86.82
Garlapati-Madras	− 19.963	1.1013	0.0044	− 5933.1954	1.2715	–	12.28	0.9607	138.75
Sparks	4.2903	0.7552	8.4564	− 22.328	–	–	11.67	0.9635	150.25
MST	− 11,606.114	144,574.41	20.4106	–	–	–	11.38	0.9608	93.3
Jouyban	8.7283	− 21.1732	− 0.000001	0.000008	− 1.5291	12.1285	14.7	0.9789	177.44
Sodeifian	− 22.5789	− 0.1889	1.9462	0.0014	0.0009	− 1050.0384	14.38	0.9472	68.04
Bartle	19.0268	− 8839.0557	0.0097	–	–	–	12.57	0.9575	85.56

Conversely, elevated temperatures may decrease the density of SC-CO_2_, reducing its overall solvating capacity. Based on Fig. 3, the pressure range of 120–150 bar corresponds to the crossover pressure zone for NHM. Density and solute vapor pressure are the dominant variables at pressures above and below the crossover pressure. At pressures under (beyond) the crossover pressure area, solubility drops (rises) with temperature. Other individuals have also reported similar findings regarding the dual effect of temperature on SC-CO_2_ solubility^60^.

**Figure 4:**
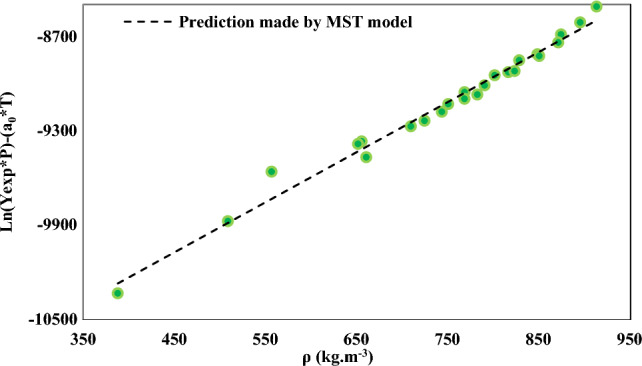
Self-consistency trend obtained by MST model, dotted line presents prediction made by MST model and symbols refer to solubility data of NHM.

Regarding the challenges in fully guaranteeing the accuracy of experimental data, their agreement with specific thermodynamic relationships can be checked to confirm their thermodynamic consistency or inconsistency. The MST model is a commonly employed thermodynamic relationship for analyzing the consistency of experimental phase equilibrium data. In addition to its correlational capacity, the potential for extrapolation is a crucial advantage of any model or correlation. As such, the Mendez-Santiago and Teja model (MST), also known as the self-consistency test (Fig. 4), was conducted to assess the extrapolative capabilities of the models under examination. The findings indicated the linear behavior of all the isotherms and isobars in this study, thus enabling the solubility results to be estimated beyond their currently calculated range^66,67^. Therefore, NHM solubility can be predicted in temperatures and pressures beyond the current range due to their simple linear behavior. The experimental data, represented by a solid line for all temperatures, were internally consistent when considering solubility.

Figure 4 demonstrates the stability of the experimental data obtained via the MST model.

### Solubility correlation

Four commonly utilized models were applied to define the correlation between the solubility data of NHM in SC-CO_2_. These models include EoS-based, modified Wilson (ELT), and density-based semi-empirical models as well as machine learning algorithms. The results were compared based on *AARD%*, R_adj_, and F-value.

#### Empirical and semi-empirical models

**Figure 5:**
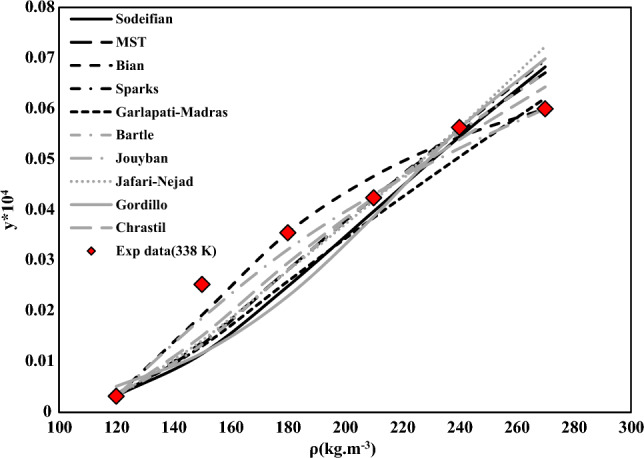
NHM solubility in SC-CO_2_. Symbols are experimental points in one temperature (338 K); various types of line are calculated with empirical and semi-empirical models.

Table 5 lists the outputs of the semi-empirical models used in this research. As shown, the mean values of *AARD%* for Chrastil, Gordillo, Sparks, Garlapati-Madras, Jafari-Nejad, Bian, Bartle, MST, Jouyban, and Sodeifian models were 12.29%, 11.67%, 12.28%, 8.11%, 12.57%, 11.38%, 14.70%, 20.92%, 11.60%, and 14.38%, respectively. Consequently, MST (R_adj_ = 0.9608, F-value = 150.25) and Bian (R_adj_ = 0.9763, F-value = 235.17) models are the best in characterizing the solubility data of NHM (some of results are summarized below in Fig. 5 at 338 K).

**Figure 6:**
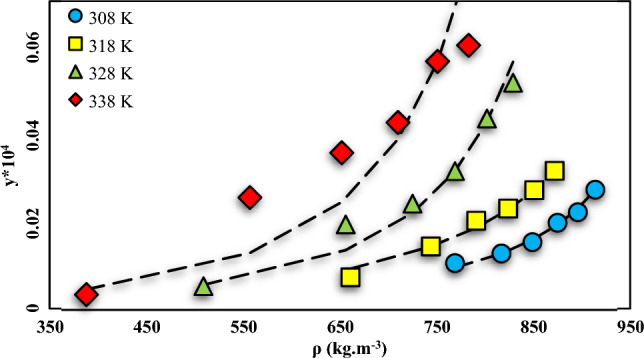
NHM solubility vs. SC-CO_2_ density. Solid lines are calculated solubilities with PR EoS model, symbols are experimental data.

**Figure 7:**
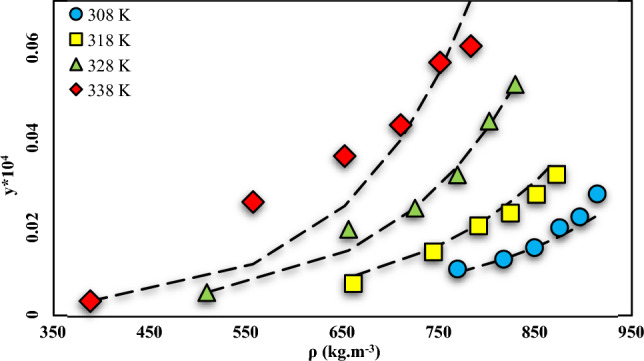
NHM solubility vs. SC-CO_2_ density. Solid lines are calculated solubilities with ELT model, symbols are experimental data.

**Figure 8:**
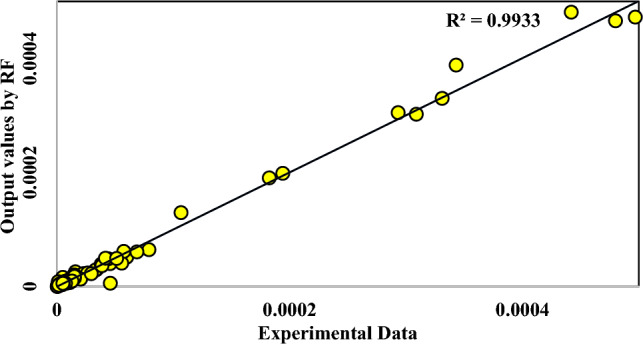
Regression line of NHM solubility (with 17 other drugs shown by symbols) vs. RF outputs.

**Figure 9:**
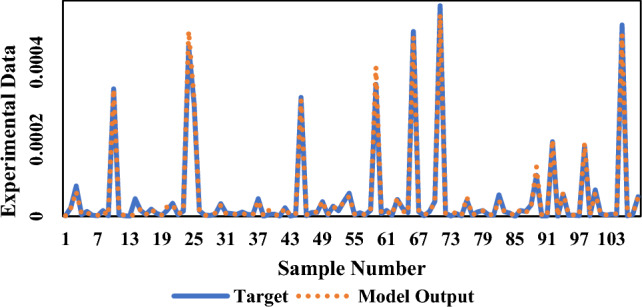
NHM solubility (with 17 other drugs shown as a solid line) vs. RF outputs shown as dots.

**Figure 10:**
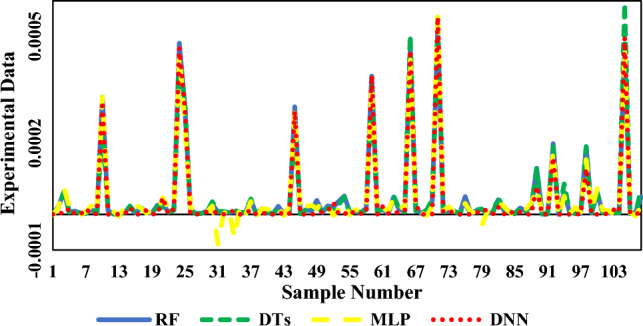
All models in one frame, lines and dots are results of models.

The models with six adjustable parameters had the lowest correlation with the experimental data, while the models with 3, 4, and 5 parameters exhibited almost the same performance in terms of correlation with the solubility data. In addition, all the models in this study demonstrated satisfactory accuracy in fitting the solubility data. Further information is available in supplementary section (Figs. 1S–10).

#### PR EoS vdW2 model

The PR EoS was utilized along with the vdW mixing rule to evaluate the correlation of solubility data at four distinct temperatures of 308, 318, 328, and 338 K. Additionally, the sublimation pressure corresponding to each temperature was modified to facilitate the comparison. For modeling the solubility data, the use of equations of state requires an initial determination of the thermodynamic characteristics of the solid substance via various methods. These characteristics entail sublimation pressure, volume at critical temperature, acentric factor, and boiling temperature. The solubility of drugs is under the direct or indirect influence of various properties. As an illustration, the solubility of a drug can be increased by elevating the sublimation pressure, which in turn raises the vapor pressure. The sublimation pressure is contingent upon the acentric factor; on the other hand, the acentric factor is highly dependent on the boiling temperature. All these factors are interdependent with a remarkable impact on the drug solubility. The Marrero-Gani and Stein Brown methods were employed to estimate the boiling temperature, while Fedors method was utilized to assess volume at critical temperature. The Ambrose-Walton method was also applied for estimation of the sublimation pressure as listed in Table 6.

**Table 6:** Evaluated critical values and physicochemical properties of NHM.

Component	T_b_ (K)	T_c_ (K)	P_c_ (bar)	ω	V_s_ (cm^3^/mol)	T (k)			
						308	318	328	338
						P_sub_^c^ (Pa)			
Nilotinib.HCl.H_2_O	725.640^a^	936.444^b^	11.77822^b^	0.5913^a^	376.8^d^	0.00040739	0.0014	0.0043	0.0125
CO_2_	–	304.18	73.8	0.274	–	–	–	–	–

^a^Estimated by Stein Brown method^68^.

^b^Estimated by Marrero and Gani method^69^.

^c^Estimated by Ambrose–Walton corresponding states method^70^.

^d^Estimated by Fedors method^71^.

According to the results in Table 7 and Fig. 6 (F-value = 119.41, R_adj_ = 0.9896), the best performance was achieved at 308 K. The correlation with the solubility data decrements with temperature enhancement and changes in the sublimation pressure. Therefore, the PR model had the lowest correlation with NHM solubility data at 338 K.

**Table 7:** Correlation outcomes for solubility of NHM in SC-CO_2_ by PR-EoS -vdW model.

Model	Parameters	T = 308 K	T = 318 K	T = 328 K	T = 338 K
PR-vdW	*K*_*12*_	0.559	0.565	0.622	0.65
	*l*_*12*_	0.568	0.561	0.649	0.687
	*AARD (%)*	3.39	6.14	9.42	26.20
	F-value	119.41	89.81	32.34	3.63
	R_adj_	0.9896	0.9862	0.9623	0.7162

The thermodynamic properties of NHM were estimated using both the Chrastil and Bartle models and the Chrastil and EoS. In this regard, vaporization (ΔH_vap_), total (ΔH_total_), and solvation (ΔH_sol_) enthalpies were calculated through the following method:

The Chrastil model (*a*_1_ = ΔH_total_/R = − 6296.2307) results in ΔH_total_ of 52.35 kJ/mol (endothermic) while Bartle model (*a*_1_ = ΔH_vap_/R = − 8839.0557) leads to ΔH_vap_ of 73.49 kJ/mol (endothermic). These computations can be expanded to estimate the solvation heat as ΔH_sol_ =|ΔH_total_ − ΔH_vap_|= 21.14 kJ/mol. Thereupon, the combination of Bartle and Chrastil models, results in the solvation enthalpy of (ΔH_sol_) − 21.14 kJ mol^−1^. Also, Table 2S (supplementary section) reports the enthalpies obtained in this work.

#### ELT model (modified Wilson model)

The correlation of modified Wilson-predicted solid–liquid equilibrium was compared with the solubility data of NHM. The results indicated that the ELT model outperformed the empirical and semi-empirical models of this work (see Table 8 and Fig. 7).

**Table 8:** Correlation outcomes for solubility of NHM in SC-CO_2_, by ELT, Modified Wilson model.

Model	α_12_ (α)	β_12_ (β)	α_21_ (λ´_12_)	β_21_ (λ´_21_)	*AARD *(%)	F-value	R_adj_
Modified Wilson	− 0.142	8.536	3.305	3.454	10.73	100.57	0.9468

#### Machine learning algorithms

In the present investigation, the solubility of NHM was studied and evaluated via different models, for the first time. Four machine-learning algorithms were also used to compare the experimental data of various drugs including 17 previously published drugs. Among these algorithms, the RF algorithm showed the highest correlation with the solubility data. According to the initial results in Table 9, the RF algorithm provided the best performance (R^2^ = 0.9933) among all tested algorithms. Figure 8 and 9 also offer more perspectives about RF model results. Figure 10 also presents the results of all models in one figure. It should be noted that supplementary figures on some algorithms can be found in supplementary section (Figs. 11S–16S).

**Table 9:** Initial results of machine learning algorithms used in this work.

Algorithm	MA_E_	MS_E_	R^2^
RF	4.33E−06	8.00E−11	0.9933
DTs	6.78E−06	2.47E−10	0.9799
MLP	1.18E−05	3.32E−10	0.9724
DNN	1.29E−05	4.44E−10	0.9701

To ensure the validity of the machine learning algorithms, two non-parametric tests were employed by Eviews13 software: (1) Augmented Dickey–Fuller (ADF) and (2) Phillips–Perron. The results indicate that the probe value is less than 1%, thereby, confirming the reliability of the models. Furthermore, as the absolute static value is higher than 1%, 5%, and 10%, the models with an error coefficient of 1% provide a sufficient degree of accuracy and precision.

1. In statistics, the ADF test is employed to evaluate the presence of unit root in a given statistical sample. The null hypothesis is tested against alternative explanations, which can include stationarity or trend-stationarity depending on the applied specific test. This test has been developed as an advanced prescription of the Dickey–Fuller test, allowing the analysis of a wider range of time-series models. The ADF statistic produced by this test is negative, and the strength of rejection for the unit root hypothesis increases as the value becomes more negative with a certain level of confidence^72^.
2. Similar to the enhanced Dickey–Fuller test, the Phillips–Perron test addresses the concern that the data producing process for a variable may exhibit a greater level of autocorrelation than that of the test equation, leading to increased endogeneity and rejection of the Dickey–Fuller t-test. The ADF test resolves this issue through including lagged variables as regressors in the test equation. In contrast, the Phillips–Perron test introduces a non-parametric alteration to the t-test statistic. This approach has shown proper robustness against nonspecific autocorrelation and heteroscedasticity within the disorder process of the test equation. Davidson and MacKinnon demonstrated that the Phillips-Perron test outperformed the augmented Dickey–Fuller test in the case of finite samples^73,74^. The ADF and Philips–Perron results are shown in Table 10.

**Table 10:** Non-parametric tests results.

Test (RF)	Adj t-stat	Test critical value			Prob	R^2^	Adj R^2^
		1% level	5% level	10% level			
ADF	− 8.590281	− 3.498439	− 2.891234	− 2.582678	0	0.9351	0.9293
Philips–Perron	− 45.54484	− 3.493747	− 2.8892	− 2.581596	0.0001	0.8147	0.813

Test (DTs)	Adj t-stat	Test critical value			Prob	R^2^	Adj R^2^
		1% level	5% level	10% level			
ADF	− 8.702216	− 3.498439	− 2.891234	− 2.582678	0	0.9379	0.9323
Philips–Perron	− 47.32203	− 3.493747	− 2.8892	− 2.581596	0.0001	0.8165	0.8148

Test (MLP)	Adj t-stat	Test critical value			Prob	R^2^	Adj R^2^
		1% level	5% level	10% level			
ADF	− 8.588913	− 3.498439	− 2.891234	− 2.582678	0	0.937	0.9314
Philips–Perron	− 49.37702	− 3.493747	− 2.8892	− 2.581596	0.0001	0.8215	0.8198

Test (DNN)	Adj t-stat	Test critical value			Prob	R^2^	Adj R^2^
		1% level	5% level	10% level			
ADF	− 8.539113	− 3.498439	− 2.891234	− 2.582678	0	0.9336	0.9277
Philips–Perron	− 45.26102	− 3.493747	− 2.8892	− 2.581596	0.0001	0.8163	0.8145

## Conclusion

Enhancing the solubility of poorly water-soluble pharmaceuticals has long been an efficient approach for producing more effective nanoparticles. Information on the solubility of drugs in supercritical conditions is highly essential to achieve this goal. This study thus explored the solubility of nilotinib hydrochloride monohydrate (NHM) in SC-CO_2_ at 308–338 K and pressure range of 120–270 bar, for the first time. The mole fraction of the drug dissolved in SC-CO_2_ ranges from 0.1 × 10^–5^ to 0.59 × 10^–5^ corresponding to the solubility range of 0.016–0.094 g/L. The maximum solubility of NHM (0.59 × 10^–5^) was achieved at 338 K and a pressure of 270 bar. The experimental data were correlated by four groups of models: (1) empirical and semi-empirical models, including Bartle (12.57%), Sodeifian (14.38%), Chrastil (12.29%), Sparks (11.67%), Galapati-Madras (12.28%), Bian (best among others with an *AARD%* of 8.11), Jouyban (14.70%), Jafari-Nejad (11.60%), MST (11.38%), and Gordillo (20.92%); (2) Peng-Robinson EoS model with vdW mixing rule (best *AARD%* in temperature of 308 K with the value of 3.39); (3) ELT (Modified Wilson model, with an *AARD%* of 10.73); and (4) machine learning techniques such as RF, DTs, MLP, and DNN (RF showed the best performance with the R^2^ value of 0.9933). The Bian and modified Wilson models exhibited the highest correlation with the experimental data. The MST model was also utilized to evaluate the self-consistency of the experimental results. Based on the correlation results proposed by Chrastil and Bartle, the NHM-CO_2_ solvation and vaporization enthalpies were estimated to be − 21.14 and 73.49 kJ/mol, respectively, that allows us to determine the thermodynamic characteristics of NHM.

### Supplementary Information

Supplementary Information.

## Data Availability

The datasets used and/or analyzed during the current study available from the corresponding author on reasonable request.

## List of symbols

AARD: Absolute average relative deviation
R_adj_: Adjusted R
M_w_: Molecular weight
T_m_: Melting point
P_sub_: Pressure of sublimation
ELT: Expanded liquid theory
SC-CO_2_: Supercritical carbon dioxide
EoS: Equation of state
SS_E_: Error sum of squares
P_c_: Critical pressure
P_r_: Reduced pressure
T_c_: Critical temperature
ADF: Augmented Dickey–Fuller
ΔH_sol_: Solvation enthalpy
ΔH_vap_: Vaporization enthalpy
y_2_: Solubility in mole fractions
a_0_–a_5_: Adjustable parameters
R: Gas constant, J/(mol K)
PR: Peng–Robinson
vdW: Van der Waals
ANN: Artificial neural network
NIST: National institute of standards and technology

## Greek symbols

Δ: Difference
μm: Micrometer
*ρ*: Density
*ρ*_*r*_: Reduced density
λ_max_: Wavelength with strongest photon absorption
ω: Acentric factor
*k*_*ij*_: EoS mixing rule parameter
*l*_*ij*_: EoS mixing rule parameter
λ_12_, λ_21_: Wilson model parameters

## Sub and superscripts

exp: Experimental
cal: Calculated
c: Critical
m: Melting
r: Reduced
sub: Sublimation
ref: Reference state

## References

[1] Wapner, J. *The Philadelphia Chromosome: A Genetic Mystery, a Lethal Cancer, and the Improbable Invention of a Lifesaving Treatment* 13–87 (The Experiment, 2014).

[2] Melnick JS (2006). An efficient rapid system for profiling the cellular activities of molecular libraries. Proc. Natl. Acad. Sci. U. S. A..

[3] Hochhaus A, La Rosée P (2004). Imatinib therapy in chronic myelogenous leukemia: Strategies to avoid and overcome resistance. Leukemia.

[4] Sodeifian G, Saadati Ardestani N, Razmimanesh F, Sajadian SA (2020). Experimental and thermodynamic analyses of supercritical CO_2_-Solubility of minoxidil as an antihypertensive drug. Fluid Phase Equilib..

[5] Sodeifian G, Bagheri H, Arbab Nooshabadi M, Razmimanesh F, Roshanghias A (2023). Experimental solubility of fexofenadine hydrochloride (antihistamine) drug in SC-CO_2_: Evaluation of cubic equations of state. J. Supercrit. Fluids.

[6] Hazaveie SM, Sodeifian G, Sajadian SA (2020). Measurement and thermodynamic modeling of solubility of Tamsulosin drug (anti cancer and anti-prostatic tumor activity) in supercritical carbon dioxide. J. Supercrit. Fluids.

[7] Sodeifian G, Alwi RS, Razmimanesh F (2022). Solubility of Pholcodine (antitussive drug) in supercritical carbon dioxide: Experimental data and thermodynamic modeling. Fluid Phase Equilib..

[8] Sodeifian G, Surya Alwi R, Razmimanesh F, Sodeifian F (2022). Solubility of prazosin hydrochloride (alpha blocker antihypertensive drug) in supercritical CO_2_: Experimental and thermodynamic modelling. J. Mol. Liq..

[9] Sodeifian G, Alwi RS, Razmimanesh F, Tamura K (2021). Solubility of Quetiapine hemifumarate (antipsychotic drug) in supercritical carbon dioxide: Experimental, modeling and Hansen solubility parameter application. Fluid Phase Equilib..

[10] Sodeifian G, Garlapati C, Razmimanesh F, Ghanaat-Ghamsari M (2021). Measurement and modeling of clemastine fumarate (antihistamine drug) solubility in supercritical carbon dioxide. Sci. Rep..

[11] Sodeifian G, Garlapati C, Roshanghias A (2022). Experimental solubility and modeling of Crizotinib (anti-cancer medication) in supercritical carbon dioxide. Sci. Rep..

[12] Sodeifian G, Razmimanesh F, Sajadian SA (2019). Solubility measurement of a chemotherapeutic agent (Imatinib mesylate) in supercritical carbon dioxide: Assessment of new empirical model. J. Supercrit. Fluids.

[13] Sodeifian G, Nasri L, Razmimanesh F, Abadian M (2021). Measuring and modeling the solubility of an antihypertensive drug (losartan potassium, Cozaar) in supercritical carbon dioxide. J. Mol. Liq..

[14] Bian X-Q, Zhang Q, Du Z-M, Chen J, Jaubert J-N (2016). A five-parameter empirical model for correlating the solubility of solid compounds in supercritical carbon dioxide. Fluid Phase Equilib..

[15] Chrastil J (1982). Solubility of solids and liquids in supercritical gases. J. Phys. Chem..

[16] Nejad SJ, Abolghasemi H, Moosavian MA, Maragheh MG (2010). Prediction of solute solubility in supercritical carbon dioxide: A novel semi-empirical model. Chem. Eng. Res. Des..

[17] Jouyban A, Chan H-K, Foster NR (2002). Mathematical representation of solute solubility in supercritical carbon dioxide using empirical expressions. J. Supercrit. Fluids.

[18] Sparks DL, Hernandez R, Estévez LA (2008). Evaluation of density-based models for the solubility of solids in supercritical carbon dioxide and formulation of a new model. Chem. Eng. Sci..

[19] Méndez-Santiago J, Teja AS (1999). The solubility of solids in supercritical fluids. Fluid Phase Equilib..

[20] Garlapati C, Madras G (2010). New empirical expressions to correlate solubilities of solids in supercritical carbon dioxide. Thermochim. Acta.

[21] Bartle KD, Clifford AA, Jafar SA, Shilstone GF (1991). Solubilities of solids and liquids of low volatility in supercritical carbon dioxide. J. Phys. Chem. Ref. Data.

[22] Gordillo MD, Blanco MA, Molero A, De La Ossa EM (1999). Solubility of the antibiotic Penicillin G in supercritical carbon dioxide. J. Supercrit. Fluids.

[23] Cortesi A, Kikic I, Alessi P, Turtoi G, Garnier S (1999). Effect of chemical structure on the solubility of antioxidants in supercritical carbon dioxide: Experimental data and correlation. J. Supercrit. Fluids.

[24] Apolloni, B., Ghosh, A., Alpaslan, F. & Patnaik, S. *Machine Learning and Robot Perception*, vol. 7 (Springer Science & Business Media, 2005).

[25] Sodeifian G, Surya Alwi R, Razmimanesh F, Abadian M (2022). Solubility of Dasatinib monohydrate (anticancer drug) in supercritical CO_2_: Experimental and thermodynamic modeling. J. Mol. Liq..

[26] Sodeifian G, Nasri L, Razmimanesh F, Nooshabadi MA (2023). Solubility of ibrutinib in supercritical carbon dioxide (Sc-CO_2_): Data correlation and thermodynamic analysis. J. Chem. Thermodyn..

[27] Sodeifian G, Behvand Usefi MM, Razmimanesh F, Roshanghias A (2023). Determination of the solubility of rivaroxaban (anticoagulant drug, for the treatment and prevention of blood clotting) in supercritical carbon dioxide: Experimental data and correlations. Arab. J. Chem..

[28] Sodeifian G, Hsieh CM, Derakhsheshpour R, Chen YM, Razmimanesh F (2022). Measurement and modeling of metoclopramide hydrochloride (anti-emetic drug) solubility in supercritical carbon dioxide. Arab. J. Chem..

[29] Ivaturi R, Sastry TM, Satyaveni S (2016). Development and validation of a stability indicating HPLC method for the determination of nilotinib hydrochloride in bulk and pharmaceutical dosage form. Int. J. Pharm. Pharm. Sci..

[30] Prausnitz, J. M., Lichtenthaler, R. N. & De Azevedo, E. G. *Molecular Thermodynamics of Fluid-Phase Equilibria* (Pearson Education, 1998).

[31] Sodeifian G, Razmimanesh F, Saadati Ardestani N, Sajadian SA (2020). Experimental data and thermodynamic modeling of solubility of Azathioprine, as an immunosuppressive and anti-cancer drug, in supercritical carbon dioxide. J. Mol. Liq..

[32] Marceneiro S, Coimbra P, Braga MEM, Dias AMA, De Sousa HC (2011). Measurement and correlation of the solubility of juglone in supercritical carbon dioxide. Fluid Phase Equilib..

[33] Cheng S-H, Yang F-C, Yang Y-H, Hu C-C, Chang W-T (2013). Measurements and modeling of the solubility of ergosterol in supercritical carbon dioxide. J. Taiwan Inst. Chem. Eng..

[34] Haghtalab A, Sodeifian G (2002). Determination of the discrete relaxation spectrum for polybutadiene and polystyrene by a non-linear regression method. Iran. Polym. J..

[35] Sodeifian G, Haghtalab A (2004). Discrete relaxation spectrum and K-BKZ constitutive equation for PVC, NBR and their blends. Appl. Rheol..

[36] Sodeifian G, Garlapati C, Razmimanesh F, Sodeifian F (2021). Solubility of amlodipine besylate (calcium channel blocker drug) in supercritical carbon dioxide: Measurement and correlations. J. Chem. Eng. Data.

[37] Sodeifian G, Garlapati C, Arbab Nooshabadi M, Razmimanesh F, Tabibzadeh A (2023). Solubility measurement and modeling of hydroxychloroquine sulfate (antimalarial medication) in supercritical carbon dioxide. Sci. Rep..

[38] Sodeifian G, Sajadian SA, Derakhsheshpour R (2020). Experimental measurement and thermodynamic modeling of Lansoprazole solubility in supercritical carbon dioxide: Application of SAFT-VR EoS. Fluid Phase Equilib..

[39] Sodeifian G, Garlapati C, Razmimanesh F, Nateghi H (2022). Experimental solubility and thermodynamic modeling of empagliflozin in supercritical carbon dioxide. Sci. Rep..

[40] Sodeifian G, Garlapati C, Razmimanesh F, Nateghi H (2022). Solubility measurement and thermodynamic modeling of pantoprazole sodium sesquihydrate in supercritical carbon dioxide. Sci. Rep..

[41] Sodeifian G, Razmimanesh F, Sajadian SA, Hazaveie SM (2020). Experimental data and thermodynamic modeling of solubility of Sorafenib tosylate, as an anti-cancer drug, in supercritical carbon dioxide: Evaluation of Wong–Sandler mixing rule. J. Chem. Thermodyn..

[42] Sodeifian G, Garlapati C, Razmimanesh F, Sodeifian F (2021). The solubility of Sulfabenzamide (an antibacterial drug) in supercritical carbon dioxide: Evaluation of a new thermodynamic model. J. Mol. Liq..

[43] Sodeifian G, Razmimanesh F, Sajadian SA (2020). Prediction of solubility of sunitinib malate (an anti-cancer drug) in supercritical carbon dioxide (SC–CO_2_): Experimental correlations and thermodynamic modeling. J. Mol. Liq..

[44] Sodeifian, G., Nasri, L., Razmimanesh, F. & Abadian, M. CO_2_ utilization for determining solubility of teriflunomide (immunomodulatory agent) in supercritical carbon dioxide: Experimental investigation and thermodynamic modeling. *J. CO2 Util.* **58**, 101931 (2022).

[45] Sodeifian G, Hsieh C-M, Tabibzadeh A, Wang H-C, Arbab Nooshabadi M (2023). Solubility of palbociclib in supercritical carbon dioxide from experimental measurement and Peng–Robinson equation of state. Sci. Rep..

[46] Somvanshi, M., Chavan, P., Tambade, S. & Shinde, S. V. A review of machine learning techniques using decision tree and support vector machine. In *2016 International Conference on Computing Communication Control and Automation (ICCUBEA)* 1–7 (IEEE, 2016). 10.1109/ICCUBEA.2016.7860040.

[47] Saghafi H, Arabloo M (2017). Modeling of CO_2_ solubility in MEA, DEA, TEA, and MDEA aqueous solutions using AdaBoost-Decision Tree and Artificial Neural Network. Int. J. Greenh. Gas Control.

[48] Breiman L (2001). Random forests. Mach. Learn..

[49] Ho, T. K. Random decision forests. In *Proceedings of 3rd International Conference on Document Analysis and Recognition*, vol. 1 278–282 (IEEE, 1995).

[50] Amit Y, Geman D (1997). Shape quantization and recognition with randomized trees. Neural Comput..

[51] Kovdienko NA (2010). Application of random forest and multiple linear regression techniques to QSPR prediction of an aqueous solubility for military compounds. Mol. Inform..

[52] Rumelhart, D. E., Hinton, G. E. & Williams, R. J. Learning internal representations by error propagation. In *Readings in Cognitive Science: A Perspective from Psychology and Artificial Intelligence* 399–421 (1988). 10.1016/B978-1-4832-1446-7.50035-2.

[53] Mahdaviara M (2021). Toward smart schemes for modeling CO2 solubility in crude oil: Application to carbon dioxide enhanced oil recovery. Fuel.

[54] LeCun Y, Bengio Y, Hinton G (2015). Deep learning. Nature.

[55] Kurotani A, Kakiuchi T, Kikuchi J (2021). Solubility prediction from molecular properties and analytical data using an in-phase deep neural network (Ip-DNN). ACS Omega.

[56] Abadian M, Sodeifian G, Razmimanesh F, Zarei Mahmoudabadi S (2023). Experimental measurement and thermodynamic modeling of solubility of Riluzole drug (neuroprotective agent) in supercritical carbon dioxide. Fluid Phase Equilib..

[57] Sodeifian G, Hazaveie SM, Sajadian SA, Saadati Ardestani N (2019). Determination of the solubility of the repaglinide drug in supercritical carbon dioxide: Experimental data and thermodynamic modeling. J. Chem. Eng. Data.

[58] Sodeifian G, Saadati Ardestani N, Sajadian SA (2019). Solubility measurement of a pigment (Phthalocyanine green) in supercritical carbon dioxide: Experimental correlations and thermodynamic modeling. Fluid Phase Equilib..

[59] Huang Z, Guo Y-H, Sun G-B, Chiew YC, Kawi S (2005). Representing dyestuff solubility in supercritical carbon dioxide with several density-based correlations. Fluid Phase Equilib..

[60] Sodeifian G, Sajadian SA, Daneshyan S (2018). Preparation of Aprepitant nanoparticles (efficient drug for coping with the effects of cancer treatment) by rapid expansion of supercritical solution with solid cosolvent (RESS-SC). J. Supercrit. Fluids.

[61] Johannsen M, Brunner G (1997). Solubilities of the fat-soluble vitamins A, D, E, and K in supercritical carbon dioxide. J. Chem. Eng. Data.

[62] Pereira PJ, Goncalves M, Coto B, de Azevedo EG, da Ponte MN (1993). Phase equilibria of CO2 + dl-α-tocopherol at temperatures from 292 K to 333 K and pressures up to 26 MPa. Fluid Phase Equilib..

[63] Span R, Wagner W (1996). A new equation of state for carbon dioxide covering the fluid region from the triple-point temperature to 1100 K at pressures up to 800 MPa. J. Phys. Chem. Ref. Data.

[64] Perrotin-Brunel H (2010). Solubility of Δ9-tetrahydrocannabinol in supercritical carbon dioxide: Experiments and modeling. J. Supercrit. Fluids.

[65] Perrotin-Brunel H (2010). Solubility of cannabinol in supercritical carbon dioxide. J. Chem. Eng. Data.

[66] Chen Y-M, Chen Y-P (2009). Measurements for the solid solubilities of antipyrine, 4-aminoantipyrine and 4-dimethylaminoantipyrine in supercritical carbon dioxide. Fluid Phase Equilib..

[67] Wang S-W, Chang S-Y, Hsieh C-M (2021). Measurement and modeling of solubility of gliclazide (hypoglycemic drug) and captopril (antihypertension drug) in supercritical carbon dioxide. J. Supercrit. Fluids.

[68] Stein SE, Brown RL (1994). Estimation of normal boiling points from group contributions. J. Chem. Inf. Comput. Sci..

[69] Marrero J, Gani R (2001). Group-contribution based estimation of pure component properties. Fluid Phase Equilib..

[70] Ambrose D, Walton J (1989). Vapour pressures up to their critical temperatures of normal alkanes and 1-alkanols. Pure Appl. Chem..

[71] Fedors RF (1974). A method for estimating both the solubility parameters and molar volumes of liquids. Polym. Eng. Sci..

[72] Fuller, W. A. *Introduction to Statistical Time Series* (Wiley, 2009).

[73] Phillips PCB, Perron P (1988). Testing for a unit root in time series regression. Biometrika.

[74] Davidson, R. & MacKinnon, J. G. *Econometric Theory and Methods*. vol. 5 (Oxford University Press, 2004).

