# Supplementary material for: A machine learning approach for thermodynamic modeling of the statically measured solubility of nilotinib hydrochloride monohydrate (anti-cancer drug) in supercritical CO2
Source: Sci Rep. 2023 Aug 9;13:12906. doi: 10.1038/s41598-023-40231-4 (PMC10412577; doi:10.1038/s41598-023-40231-4)
Supplement: Supplementary file 1 — Supplementary Information. [file 41598_2023_40231_MOESM1_ESM.pdf]

Supplementary information

A machine learning approach for thermodynamic modeling of the statically measured solubility of nilotinib hydrochloride monohydrate (anti-cancer drug) in supercritical CO_2

Hassan Nateghi [1,2,3], Gholamhossein Sodeifian [1,2,3 *], Fariba Razmimanesh[1,2,3], Javad Mohebbi Najm Abad [4]

[1] Department of Chemical Engineering, Faculty of Engineering, University of Kashan, 87317-53153, Kashan, Iran

[2] Laboratory of Supercritical Fluids and Nanotechnology, University of Kashan, 87317-53153, Kashan, Iran

[3] Modeling and Simulation Centre, Faculty of Engineering, University of Kashan, 87317-53153, Kashan, Iran

[4] Department of Computer Engineering, Quchan Branch, Islamic Azad University, 9479176135, Quchan, Iran

*Corresponding author. Tel.: +983155913393; fax: +983155912424.

Email address: sodeifian@kashanu.ac.ir ***(G. Sodeifian)***

1S. Introduction

NHM is known to treat certain types of chronic myelogenous leukemia, a form of bone marrow-derived white blood cell cancer. The phrase "chronic" refers to the idea that the disease progress is slower than the acute type, while the term "myeloid" is associated with a group of malignant bone marrow cells with irregular and extensive expansion. Chronic myeloid leukemia is more common in the middle-aged and older population, with a rare incidence among children. However, cases of pediatric patients are documented as well. Moreover, NHM is a promising alternative in cases where the patient did not respond to imatinib or cannot tolerate its side effects. NHM belongs to a family of medicines known as enzyme inhibitors. It can be also employed to treat children above the age of one who do not respond to existing enzyme inhibitor medications or cannot take these drugs due to their toxicity. NHM inhibits an atypical protein from activating the proliferation of cancerous cells. This process assists in the prevention or deceleration of the malignancy expansion.

2S. EoS-based model

Table 1S presents a summary of the EoS-based model.

Table 1S. Summary of cubic EoS model applied in this research.

Name	Formula	a	b
Peng–Robinson (PR)	$P = \frac{RT}{v-b} - \frac{a(T)}{v(v+b)+b(v-b)}$	$0.45724\frac{R^2T_c^2}{P_c} \times a\left(T_{r,}\omega\right)$	$0.07780\frac{RT_c}{P_c}$

Consequently, a suitable EoS and a definite mixing rule (e.g., vdW) are utilized to determine the fugacity coefficient of solid solute in the fluid phase, also represented by ϕ_2^s [34]:

$$\ln\left(\phi_s^f\right) = \frac{1}{RT}\int_{\infty}^{\upsilon}\left[\left[\frac{\partial P}{\partial n_2}\right]_{T,\upsilon,n_1} - \frac{RT}{\upsilon}\right]d\upsilon - \ln z \tag{1}$$

The estimation of $\phi_2^{scCO_2}$ is performed using the PR-EoS in conjunction with vdW2, where n_2 represents the moles of solute, n_1 denotes the moles of CO_2, and υ represents the molar volume of the mixture.

3S. ELT model

Prausnitz et al. presented the equation for activity coefficient (γ_2), solid solubility (y_2) in mole fraction, and fugacity of the pure solid solute (f_2^{oL}) in the expanded liquid phase [38,39]:

$$\ln\left(\frac{f_2^{os}}{f_2^{oL}}\right) = \frac{-\Delta H_2^f}{R}\left(\frac{1}{T} - \frac{1}{T_m}\right) - \frac{\Delta c_p}{RT}\left(\frac{T-T_m}{T}\right) + \frac{\Delta c_p}{R}\ln\left(\frac{T}{T_m}\right) \tag{2}$$

The heat capacity terms in the above equation can be ignored to a reasonable degree. Combining Eqs. (2), and (3), an equation can be derived for the solute solubility:

$$y_2 = \frac{1}{\gamma_2^{\infty}}\exp\left(\frac{-\Delta H_2^f}{R}\left(\frac{1}{T} - \frac{1}{T_m}\right)\right) \tag{3}$$

The enthalpy of fusion, melting point temperature of the solid solute, and activity coefficient of the solid solute at infinite solution are represented by $-\Delta H_2^f$, T_m, and γ_2^{∞}, respectively.

This includes a combinational contribution relying on Flory's theory and the value of the Gibbs excess energy [38].

$$\frac{g^E}{RT} = -\sum_i x_i \ln\left(\sum_j x_j \Lambda_{ij}\right) \tag{4}$$

where g^E is the excess Gibbs energy, and Λ_{12}, and Λ_{21} are adjustable parameters.

$$ln\gamma_i = -\ln\left(\sum_j x_j \Lambda_{ij}\right) + 1 - \sum_k \frac{x_k \Lambda_{ki}}{x_j \Lambda_{kj}} \tag{5}$$

Based on the theory of Assael et al. [39], Eq. (5) can be simplified for infinite dilution cases:

$$ln\gamma_2^{\infty} = 1 - \Lambda_{12} - ln\Lambda_{21} \quad (6)$$

Where Λ_{12} and Λ_{21} are demonstrated at infinite dilution situation:

$$\Lambda_{12} = \upsilon_2 \rho_c \rho_r \exp\left(-\frac{\acute{\lambda_{12}}}{T_r}\right) \quad (7)$$

$$\Lambda_{21} = \frac{1}{\upsilon_2 \rho_c \rho_r} \exp\left(-\frac{\acute{\lambda_{21}}}{T_r}\right) \quad (8)$$

The reduced density of the SCF is denoted by ρ_r ($\rho_r = \frac{\rho}{\rho_c}$), whereas ρ_c represents the critical density. The molar volume of the solid solute is represented by υ_2.

3S. Semi-empirical models

$$R^2 = 1 - \frac{SS_E}{SS_T} \quad (9)$$

SS_E shows the error of squares summation, while SS_T indicates the total of squares summation.

4S. Random Forests (RF)

The Gini index techniques evaluate the subset of characteristics picked in each inner node. The division feature in that node is determined based on the element with the most extraordinary Gini index. The Gini index [56] was formulated by Breiman, Friedman, Olshen, and Stone to assess data purity or confidence in the occurrence of an event that would determine the classification label. However, it should be noted that the index was originally developed in 1912 by Corrado Gini, an Italian statistician. In its most basic form, Gini index can be computed by:

$$Gini(t) = 1 - \sum_{i=1}^{N} P\left(\frac{C_i}{t}\right)^2 \quad (10)$$

In which, *t* represents a specific situation. *N* denotes the total number of classes in the data set under examination and C_i refers to the tag assigned to each class within the data set.

The estimations are detailed in Table 2S.

Table 2S. Evaluated values of total (Δh_{total}), vaporization (Δh_{vap}), and solvation (Δh_{sol}) enthalpies of NHM.

Compound	ΔH_{total} (kJmol^{-1})	ΔH_{vap} (kJmol^{-1})	ΔH_{sol} (kJmol^{-1})
Nilotinib.HCl.H_2O	52.35	73.49	-21.14

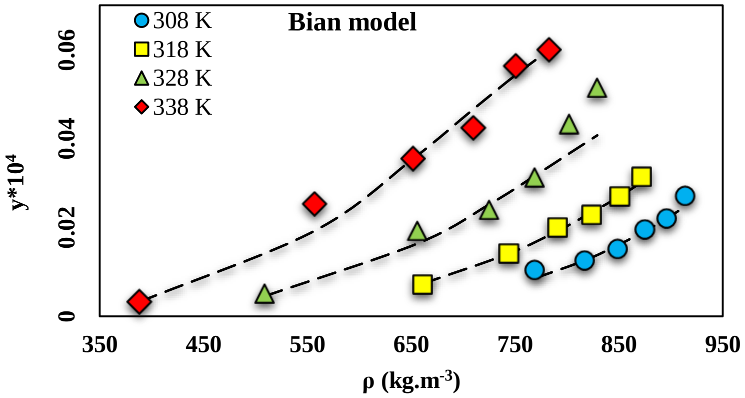

Fig. 1S. NHM solubility in SC-CO_2. Symbols are experimental points and lines are calculated with Bian model.

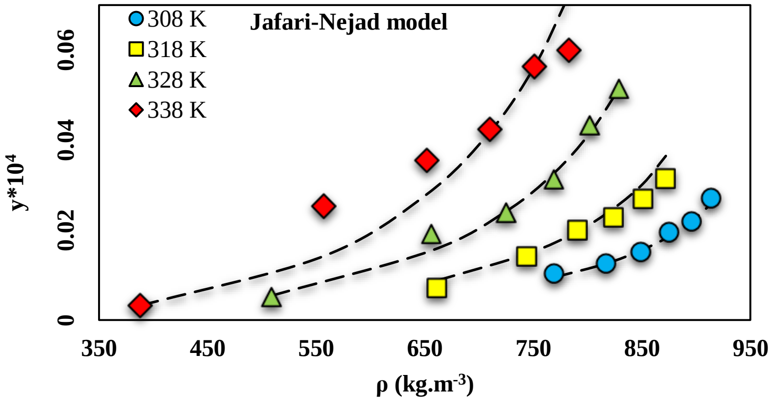

Fig. 2S. NHM solubility in SC-CO_2. Symbols are experimental points and lines are calculated with Jafari-Nejad model.

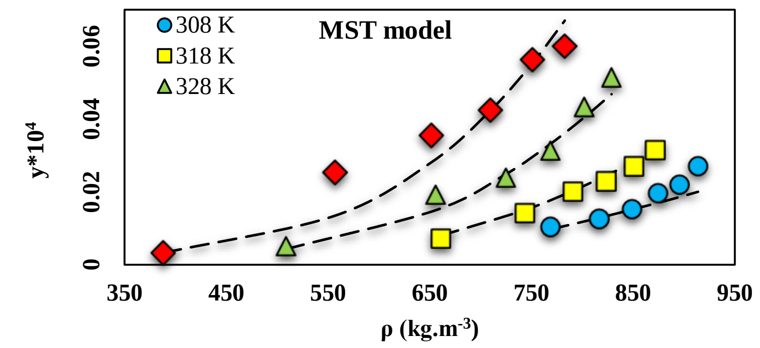

Fig. 3S. NHM solubility in SC-CO_2. Symbols are experimental points and lines are calculated with MST model.

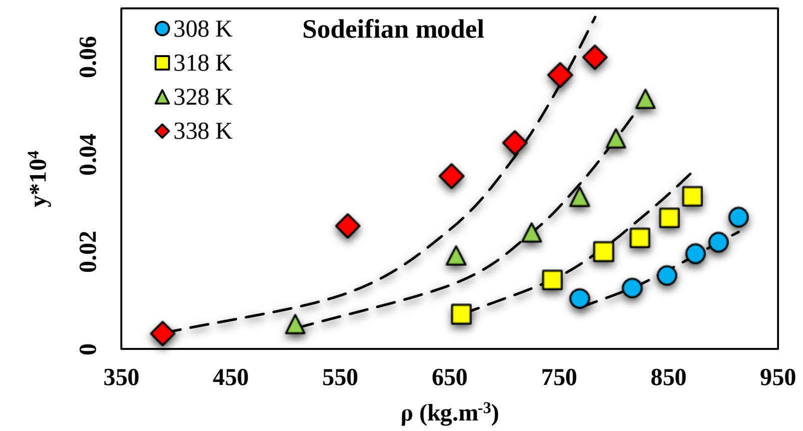

Fig. 4S. NHM solubility in SC-CO_2. Symbols are experimental points and lines are calculated with Sodeifian model.

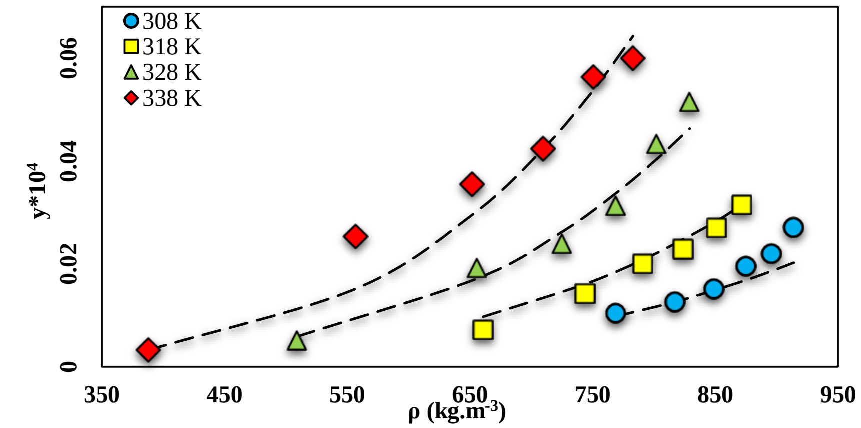

Fig. 5S. NHM solubility in SC-CO_2. Symbols are experimental points and lines are calculated with Chrastil model.

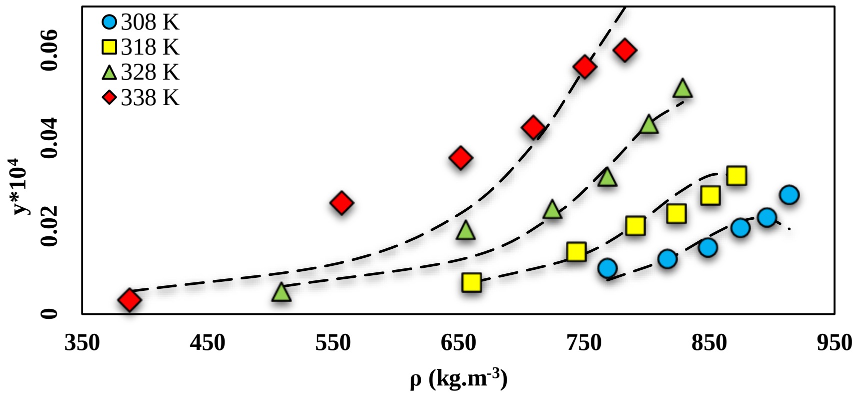

Fig. 6S. NHM solubility in SC-CO_2. Symbols are experimental points and lines are calculated with Gordillo model.

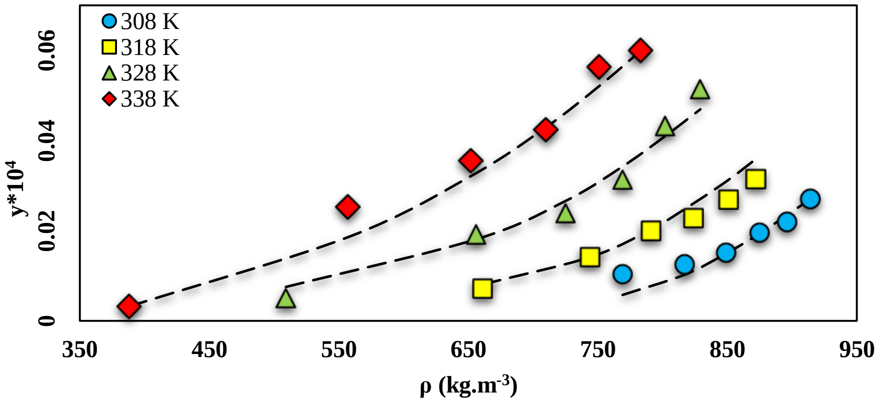

Fig. 7S. NHM solubility in SC-CO_2. Symbols are experimental points and lines are calculated with Jouyban model.

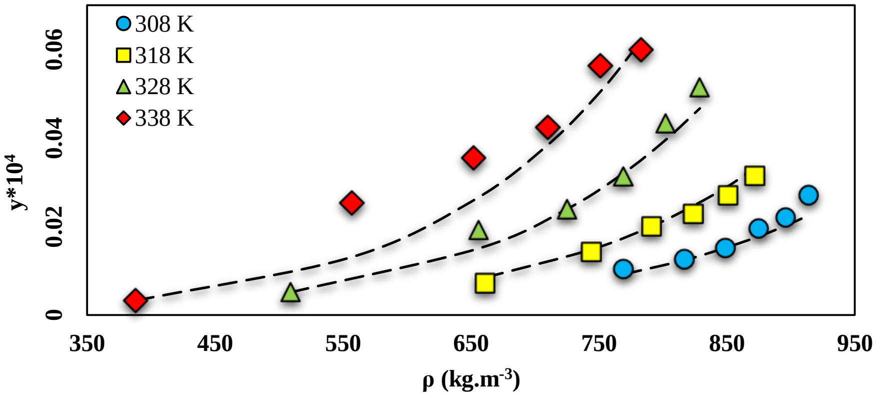

Fig. 8S. NHM solubility in SC-CO_2. Symbols are experimental points and lines are calculated with Garlapati-Madras model.

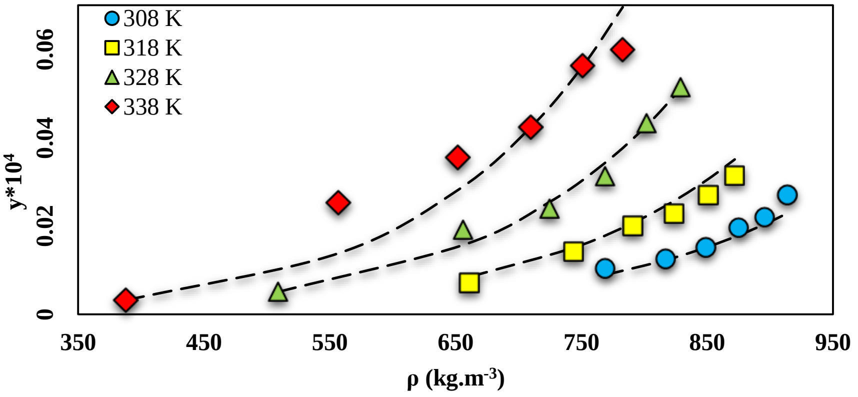

Fig. 9S. NHM solubility in SC-CO_2. Symbols are experimental points and lines are calculated with Sparks model.

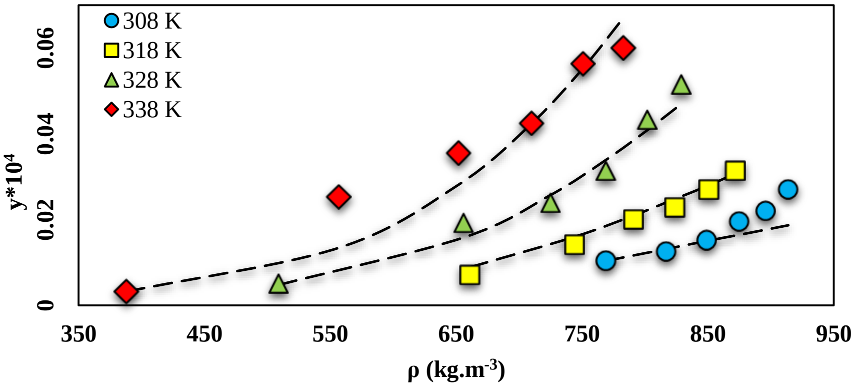

Fig. 10S. NHM solubility in SC-CO_2. Symbols are experimental points and lines are calculated with Bartle model.

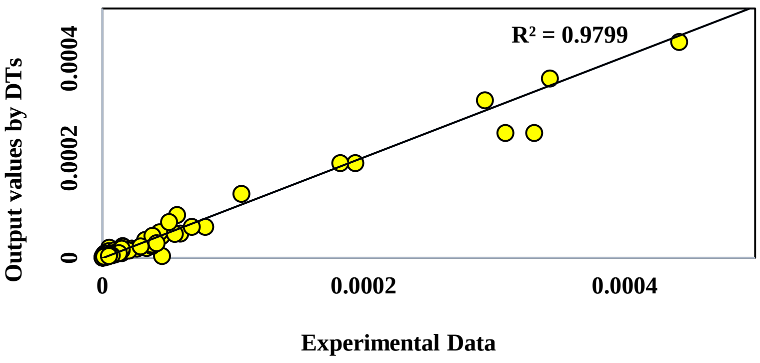

Fig. 11S. Regression line of NHM solubility (with 17 other drugs shown by symbols) vs. DTs outputs

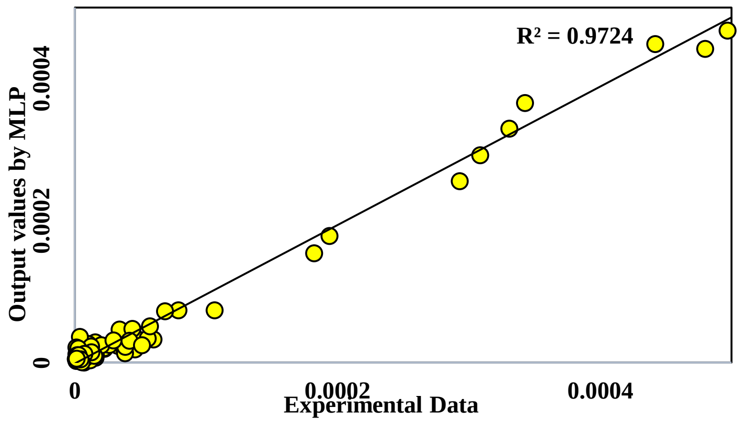

Fig. 12S. Regression line of NHM solubility (with 17 other drugs shown by symbols) vs. MLP outputs

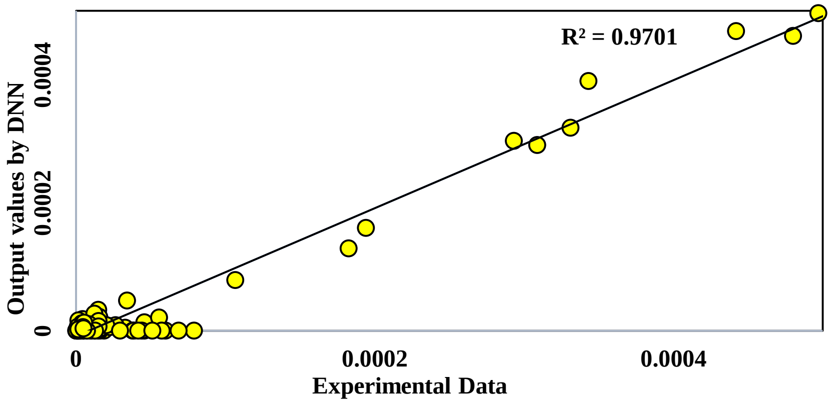

Fig. 13S. Regression line of NHM solubility (with 17 other drugs shown by symbols) vs. DNN outputs

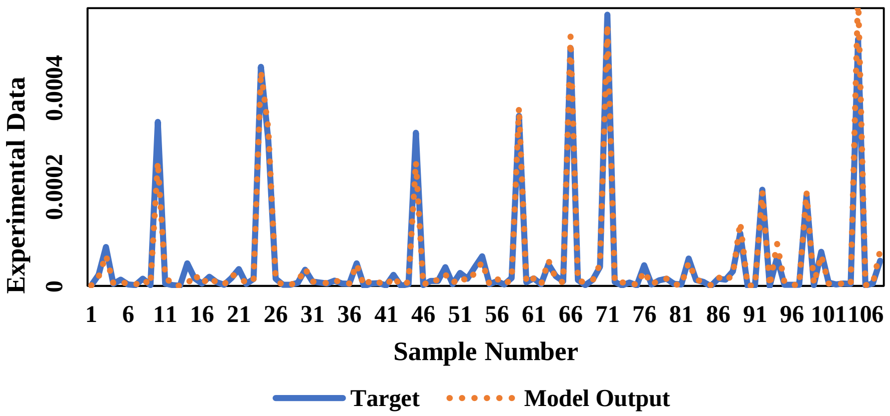

Fig. 14S. NHM solubility (with 17 other drugs shown as a solid line) vs. DTs outputs shown as dots.

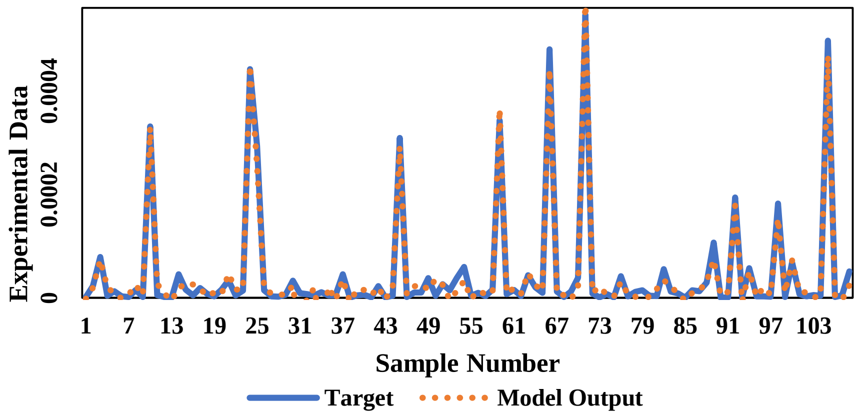

Fig. 15S. NHM solubility (with 17 other drugs shown as a solid line) vs. MLP outputs shown as dots.

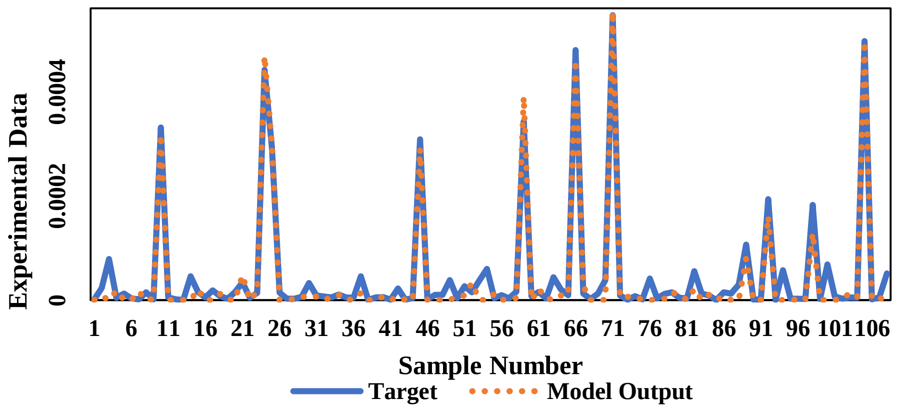

Fig. 16S. NHM solubility (with 17 other drugs shown as a solid line) vs. DNN outputs shown as dots.

.
